# Theoretical and experimental study of dual-fiber laser ablation for prostate cancer

**DOI:** 10.1371/journal.pone.0206065

**Published:** 2018-10-24

**Authors:** Xing Wu, Kangwei Zhang, Yini Chen, Ren Wang, Lei Chen, Aili Zhang, Bing Hu

**Affiliations:** 1 Department of Ultrasound in Medicine, Shanghai Jiao Tong University Affiliated 6th People’s Hospital, Shanghai, China; 2 Shanghai Institute of Ultrasound in Medicine, Shanghai, China; 3 School of Biomedical Engineering, Shanghai Jiao Tong University, Shanghai, China; University of Maryland, UNITED STATES

## Abstract

Single-fiber laser treatment of the prostate has been widely accepted in the clinic due to its minimal invasiveness and high controllability. However, for large tumors, multiple insertions of the laser probe would be needed to achieve full coverage of the tumor, increasing the complexity of the treatment and occasionally resulting in the incomplete killing of tumor cells due to a mismatch between the planned insertion location and the actual probe insertion location. Treatment with a dual-fiber laser results in greater lesion coverage following a single insertion of the probe, with the lesion coverage being even greater than the sum of the coverage of two sequential insertion of a single-fiber laser probe, potentially reducing treatment time and clinical complications. Both theoretical and experimental analyses have been performed to evaluate the proposed dual-fiber laser treatment. A finite element model was established to simulate the treatment process. The simulation results indicated that there is a clear difference between the ablation coverage created using dual-fiber laser ablation and that created using the superposition of sequential single-fiber laser ablation. In addition, the coverage is dependent on the spacing distance between the two fibers. Both *ex vivo* and *in vivo* canine prostate tissues were treated by dual-fiber laser ablation, with lesions analyzed by magnetic resonance imaging (MRI), ultrasound imaging, and pathology. The results demonstrate that dual-fiber laser ablation can markedly increase the range of the ablation zone when compared with single-fiber modes. The safety and feasibility of dual-fiber laser treatment has been confirmed, and a treatment plan using dual-fiber laser ablation has also been proposed.

## 1. Introduction

Laser ablation, which allows for concentrated energy pulses and precise control over the thermal field, has been widely accepted in clinical treatments of prostate cancer [1–4]. Studies on laser ablation in prostate cancer have included clinical trials for low-grade human prostate cancer, experimental investigations in the canine prostate, and theoretical models in the rat prostate [5–7]. Among these studies, a single-fiber laser has been used to create ablation lesions with clear local boundaries around a target by moving the fiber to multiple positions under the guidance of surgical imaging techniques. Such procedures induce large-scale tissue damage by creating multiple lesions. However, because the ablation process creates many bubbles, physicians must wait approximately 15 minutes before re-positioning the laser probe to produce a second lesion, which significantly extends the duration of surgery. Most importantly, cancer cells may survive in the margins between adjacent lesions.

By comparison, multiple laser fibers can be used (e.g., a dual-fiber laser source), and due to the interactions of the two fibers, the resulting lesion volume will be much larger than the pure superposition of two single-fiber ablations. When planning for serial ablative lesion using single-fiber probe treatments, the second lesion must be adjusted on the basis of the previous lesion which is difficult to image using current techniques. In contrast, during dual-fiber laser treatment, the second laser probe is inserted relative to the first laser probe, which can be clearly imaged. Thus, for targeted prostate cancer tissues larger than a single-fiber ablative lesion, the use of dual fibers may greatly decrease the duration of surgery and the risk of complications.

Due to the complex physiological environment in which the prostate is located, careful pretreatment planning is crucial to the final outcome of prostate cancer treatment. Several theoretical models have been proposed to simulate laser ablation for better clinical planning. S.C. Jiang has reported a simulation of laser thermotherapy and discussed the dependence of thermal damage on laser wavelength, power, duration, and other factors [8]. Paola Saccomandi and others have proposed the use of Gaussian distributions and the Beer-Lambert law to describe the distribution of laser power [9–10]. F. M. Di Matteo et al. has confirmed, through several experiments, that numerical simulation results based on the same model agree well with the experimental results [11], suggesting that numerical simulations can be used to guide laser ablation treatments. However, the aforementioned studies do not take the synergistic effect of multiple optical fibers into account, and the outcome of dual-fiber laser ablations remains unknown.

Therefore, to realize a more efficient and minimally invasive treatment of large prostate cancer, the dual-fiber laser is proposed in this study. Both finite element simulations and canine prostate ablation experiments are performed to investigate the feasibility of the proposed treatment. A model has been established to predict the treatment outcome and to examine the effects of the distances between the two fibers of the laser. Both *ex vivo* and *in vivo* canine prostate dual-fiber ablation experiments are performed, and the lesion volumes are studied using MRI, ultrasound imaging, and histological analysis.

## 2. Materials and methods

### 2.1 Theoretical model

Heat transfer in prostate tissue is described by the well-known Pennes bioheat transfer equation [5,8–10,12–14]:

$$\rho ∙c\frac{\partial T(x,y,z,t)}{\partial t}=\nabla ∙(k\nabla T(x,y,z,t))+Q_{blood}+Q_{met}+Q_{laser}-Q_{e}$$ (1)

where ρ, c, and k are the density (kg/m^3^), specific heat capacity (W/m^3^ ∙ K), and thermal conductivity (W/m ∙ K) of prostate tissue, respectively, and T(x,y,z,t) is the tissue temperature. The patterns in Eq (1) are defined below:

(1) Q_blood_ (W/m^3^) is the heat transfer resulting from blood perfusion, which can be calculated as:

$$Q_{blood}=\omega_{b}\rho_{b}c_{b}∙(T-T_{b})$$ (2)

The blood perfusion rate of prostate tissue (ω_b_) was set to be 0.0056 1/s [15] for *in vivo* tissue and 0 1/s for *ex vivo* tissue.

(2) Q_met_ (W/m^3^) is the metabolically generated heat. The transient heat flux produced by the laser is extremely large and the heat flux resulting from metabolism is very small. Therefore, the metabolic heating rate was neglected, i.e., *Q*_*met*_ = 0.

(3) Q_e_ (W/m^3^) is the energy change due to water evaporation and can be expressed by the following equation:

$$Q_{e}=-h_{fg}∙\frac{d\rho_{w}}{dt}$$ (3)

where $h_{fg}$) (J/kg) is the latent heat for water evaporation, and ρ_w_ denotes the water content of the tissue (kg/m^3^). Assuming that all water, including water vapor, remained in the prostate tissue, ρ_w_ can be expressed as follows [9–10]:

$$\rho_{w}(T)=\left\{ \begin{array}{l} \;0.778∙(1-e^{\frac{T-106}{3.42}})\;T\leq 103\\ 0.0289∙T^{3}-8.924∙T^{2}+919.6T-31573\;103\leq T\leq 104\\ \;0.778{∙e}^{\frac{T-80}{34.37}}\;T\geq 104 \end{array}\right.$$ (4)

Q_e_ can be expressed as the following form:

$$Q_{e}=-h_{fg}∙\frac{\partial \rho_{w}}{\partial T}∙\frac{\partial T}{\partial t}$$ (5)

As a result, in Eq (1), an effective specific heat capacity can be used to represent the effect of water evaporation:

$$c^{\prime }=c-\frac{h_{fg}}{\rho }\frac{\partial \rho_{w}}{\partial T}$$ (6)

Therefore, Eq (1) can be re-written in the following form:

$$\rho ∙c\prime ∙\frac{\partial T(x,y,z,t)}{\partial t}=\nabla ∙(k\nabla T(x,y,z,t))+Q_{blood}+Q_{laser}$$ (7)

(4) Q_laser_ (W/m^3^) denotes the laser power absorbed by tissues and can be described by the Beer-Lambert law [11]:

$$Q_{laser}=\alpha ∙I(x,y)∙e^{-\alpha z}$$ (8)

where I(x,y)(W/m^2^) denotes the power distribution of the laser along the cross section of the incident direction and can be described by a two-dimensional Gaussian distribution. *σ* = *r*_*f*_/3 was used to ensure that 99% of the laser power was concentrated on the cross section of the optical fiber core (r_f_ is the radius of the optical fiber):

$$I(x,y)=I_{0}∙e^{-(x^{2}+y^{2})/2\sigma^{2}}$$ (9)

where *I*_0_ = *P*/(2 ∙ *π* ∙ *σ*^2^). P(W) is the laser power in continuous output mode.

In Eq (8), the Beer-Lambert law describes the absorption of laser power along the z direction; α denoted the absorption coefficient (m^−1^), which is dependent on the laser wavelength and tissue properties. As the scattering coefficient in tissues was large, it could not be ignored; thus, an effective attenuation coefficient α_eff_ was used to replace α in Eq (8):

$$\alpha_{eff}=\sqrt{3\alpha (\alpha +\alpha_{s}(1-g))}$$ (10)

where *g* is the anisotropic coefficient and α_s_ is the scattering coefficient (m^−1^). The variables α, α_s_ and *g* are temperature dependent, due to tissue denaturation, and values corresponding to the temperature range were applied.

Eq (1) can be solved using finite element analysis software; the parameters in the model were summarized in Table 1. To investigate the properties of the heating process in dual-fiber laser ablation, two optical fibers were implanted into the tissue in parallel. Four different spacing distances between the optical fibers (3, 6, 9, and 12 mm) were evaluated. The initial temperature of the *in vivo* tissue was 37°C, the initial temperature of the *ex vivo* tissue was 20°C, and the external boundary condition was set at a constant temperature (20°C). In this study, a laser power of 5 W was applied with duration of 100 s, which was commonly used in clinic. The finite element numerical method was applied, and all the simulations were carried out in COMSOL Multiphysics 5.2.

**Table 1:** Physical parameters used in the dual-fiber laser ablation model.

Parameters	Units	Numerical Values	References
***ρ***	kg/m^3^	1079	[12]
***c***	W/m^3^ ∙ K	4000	[12]
***k***	W/m ∙ K	0.52	[12]
***ρ***_***b***_	kg/m^3^	1000	[12]
***c***_***b***_	W/m^3^ ∙ K	4180	[12]
***α***	m^−1^	30 (T<65°C); 40 (T>65°C)	[13]
***α***_***s***_	m^−1^	8000 (T < 65°C); 18000 (T > 65°C)	[13]
***g***		0.95	[13]

### 2.2 Experimental evaluation

This research was conducted in accordance with the guidelines of the National Institutes of Health of China for the care and use of laboratory animals. All of the experimental protocols were approved by the local animal ethics committee affiliated with Shanghai Sixth People’s Hospital.

#### 2.2.1 Experimental methods for *ex vivo* canine prostate tissue

**Fig 1:**
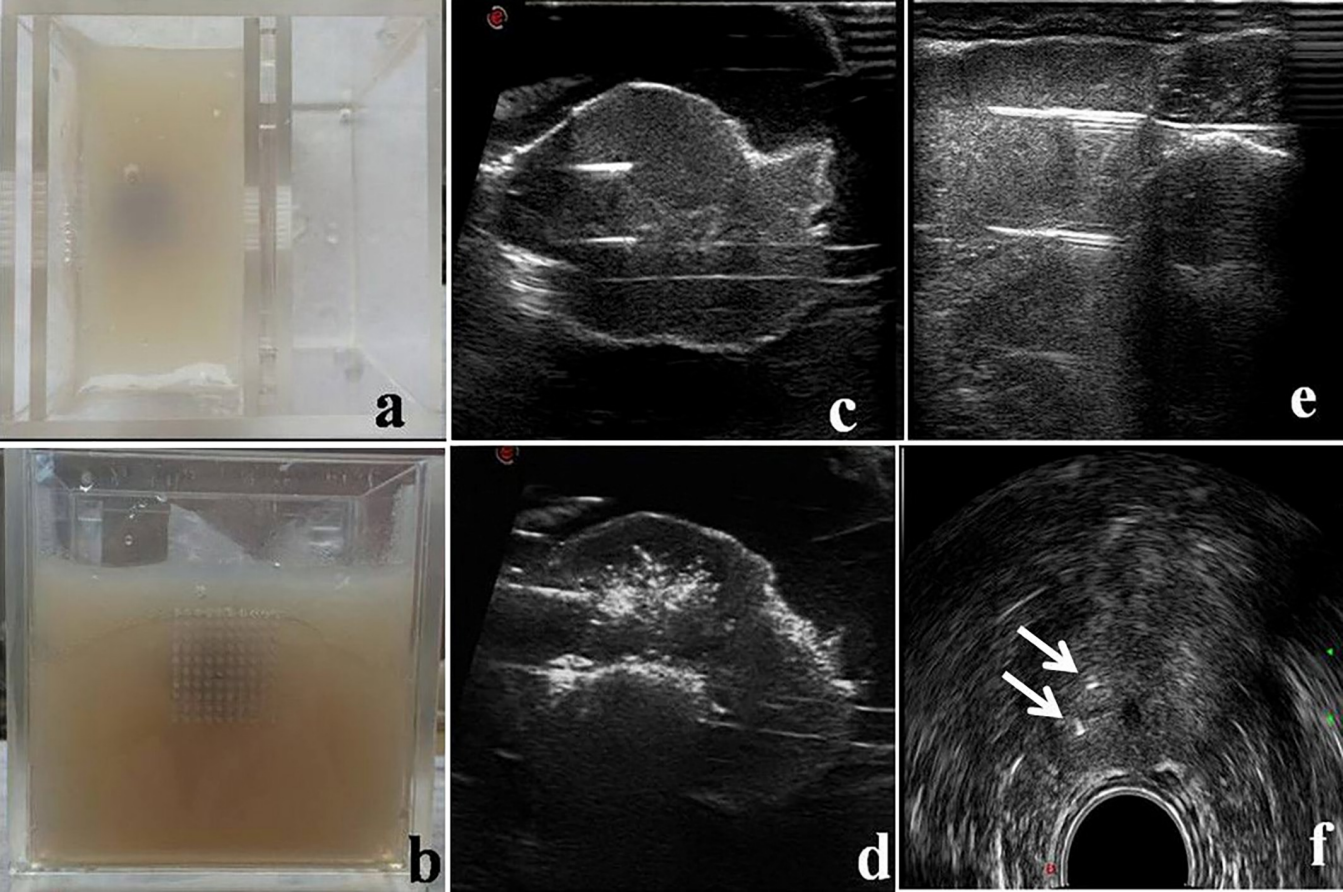
*Ex vivo* prostate placement and optical fibers arrayed in *ex vivo* and *in vivo* experiments. (a) The prostate was sealed in a box filled with agar. (b) One side of the box consisted of a guide-groove substrate. (c) The positions of the two optical fibers were monitored using ultrasound in *ex vivo* experiments. (d) The ablation process revealed a large number of strong-echo light spots. (e) The positions of the two optical fibers were monitored using ultrasound in *in vivo* experiments. (f) The cross section of the short axis revealed the biopsy needles as two strong point echoes (white arrow).

Three 5-year-old beagle dogs were euthanized by injections of potassium cyanide (KCN) under general anesthesia, and the prostate was excised. Each individual *ex vivo* canine prostate was embedded in 2% agar and placed in a custom-made acrylic box. One side of the box served as a guide-groove substrate, which was used to position the optical fibers. The guide-groove substrate consisted of two plates, on each of which 10×10 mesh holes with diameters of approximately 1.5 mm were evenly distributed, with 3-mm spacing between holes (Fig 1A and 1B).

Under ultrasound guidance, two optical fibers were inserted into one lateral lobe of the *ex vivo* prostate; the two laser fibers were parallel, the insertion depth was 12 mm, and the distance between the top and bottom fibers was 6 mm (Fig 1C). The laser therapeutic system used was an Echolaser X4 emitting a Nd: YAG laser set at a wavelength of 1064±10 nm. The length and diameter of the laser fiber used were 15 cm and 300 μm, respectively, and the external diameter of the equipped guided percutaneous needle was 18 G. The power and energy output of each laser fiber were 5 W and 500 J, respectively. The Mylab Twice ultrasound interventional system (5 MHz ultrasound) was used to monitor the ablation process (Fig 1D). After ablation, the prostate tissues were dissected along the puncture needle. The section was considered to be the largest ablation plane.

#### 2.2.2 Experimental methods for *in vivo* canine prostate tissue

In this experiment, four 5-year-old beagle dogs were used. The following experimental procedures were performed on each dog after anesthesia by an intravenous injection of sodium pentobarbital at 30 mg/kg. Among the four dogs, three were ablated by double laser fibers (A-D), and one by single fiber ablation (E). Under the guidance of a TRT23-type biplane transrectal ultrasound probe, the fibers were inserted into the right lobe of the prostate after transrectal biopsy; the two fibers were parallel with a spacing distance of 7 mm (Fig 1E and 1F). The power of a single optical fiber was 5 W, and the energy output was 500 J. The vital signs of the canines were closely monitored throughout the ablation process.

After treatment, an enhanced dynamic MRI scan was performed after an intravenous bolus injection of the contrast agent, gadolinium-diethylenetriamine Penta-acetic acid (Gd–DTPA), at 0.1 mmol/kg. A SIGNA-type superconductive MRI from GE was used; the magnetic field strength was 1.5 T, and the coil used was a surface coil. After the MRI scan, the dogs rested for 30 min and then underwent transrectal prostatic contrast-enhanced ultrasound sonography (CEUS). A TRT23-type biplane transrectal ultrasound probe (Esaote, Italy) was used; the ultrasound used was a Mylab Twice (Esaote, Italy). An intravenous bolus injection was used to inject the contrast agent, Sonovue (Bracco, Italy).

The dogs were put in general anesthesia during the whole process (about one and a half hours), and no sign of stress was observed. After experiments, all dogs were immediately euthanized by the injection of potassium cyanide (250mg) under general anesthesia, and the prostate was excised for the observation of immediate effects by ablation. The right lobe of the prostate was longitudinally sliced along the needle direction; one slice was obtained every 3 mm.

## 3. Results

### 3.1 Simulation results

**Fig 2:**
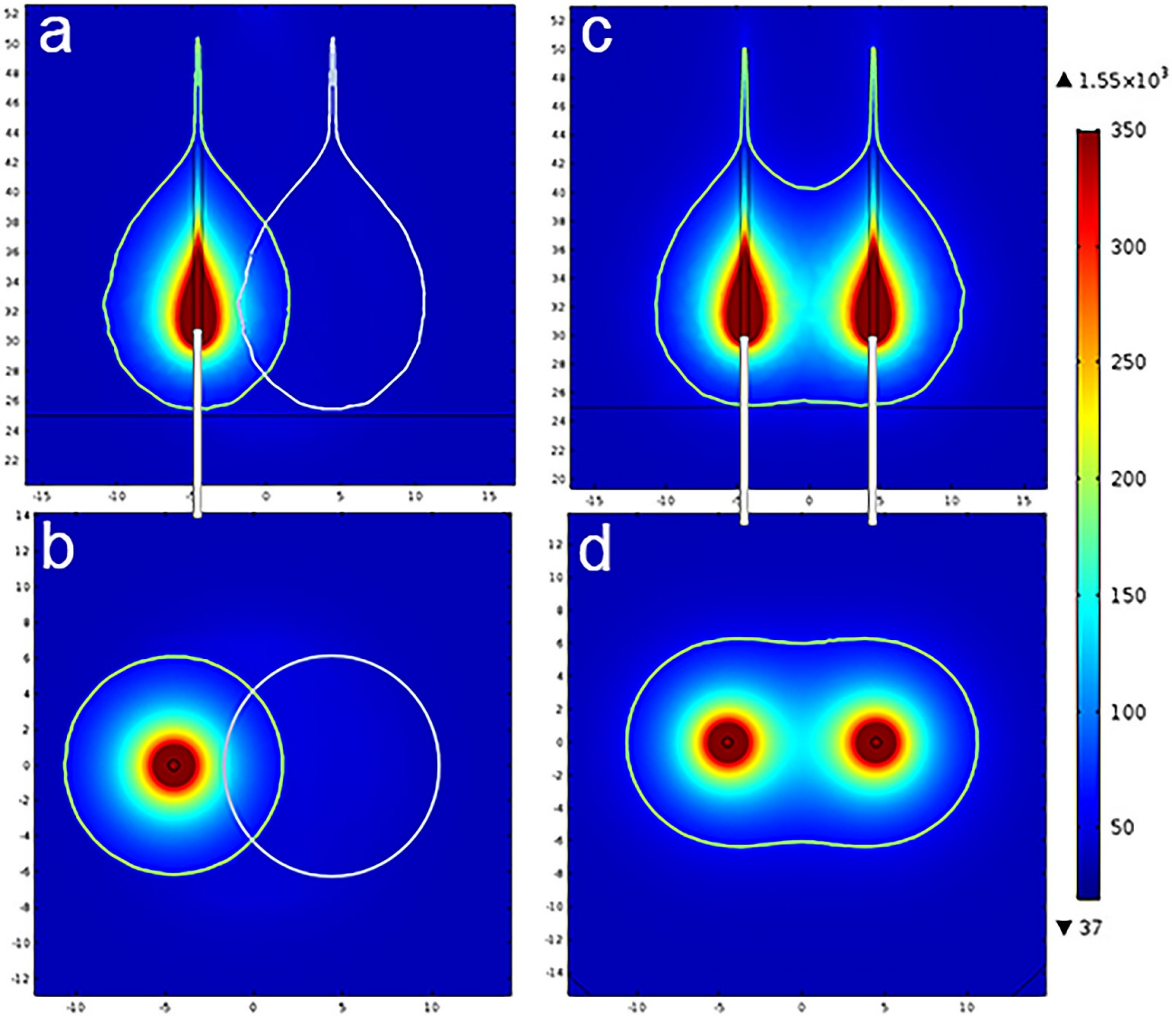
Temperature distributions after heating for 100 s with single- and dual-fiber lasers. The isothermal temperature is 60°C; the red region in the center has a temperature greater than 350°C. (a, c) Temperature distributions in the maximum ablation plane parallel to the plane of the fibers; (b, d) Temperature distributions of the maximum ablation plane perpendicular to the plane of the fibers; (a, b) The ablation region from two serial single-fiber ablations with a spacing distance of 9 mm, as indicated by the dotted line. (c, d) The ablation region of dual-fiber laser ablation with a spacing distance of 9 mm.

The simulated temperature distribution with either a single fiber or dual fibers is shown in Fig 2. A critical temperature (i.e. 60°C) was used to define the boundary of tissue necrosis [14–17]. Fig 2A and 2B illustrated the temperature profiles for two serial single-fiber laser treatments in a central cross-sectional plane parallel and perpendicular to the laser fiber. A single-fiber laser lesion exhibited a drop-like shape, consistent with the findings of other studies [9–10]. The serial fibers were spaced 9 mm apart. The total superposed lesion is illustrated by a dotted line in Fig 2A and 2B. As seen in Fig 2A, the majority of the area between the two fibers was not within the ablated range. In contrast, in a lesion created using a dual-fiber laser with the same spacing of 9 mm, a much larger ablated region was created between the two fibers, particularly in the area closer to the distal end of the laser, as shown in Fig 2C and 2D, and the lesion exhibited a kettle-like shape. The tissue volume with a temperature higher than 60°C defined as ablated region can be directly calculated by the “Volume Integration” function of COMSOL. The total ablated region produced by two serial single-fiber treatments was approximately 3.8 × 10^−6^ *m*^3^, while that of the dual-fiber treatment was approximately 4.85 × 10^−6^ *m*^3^ when the fibers were spaced 6 mm apart.

**Fig 3:**
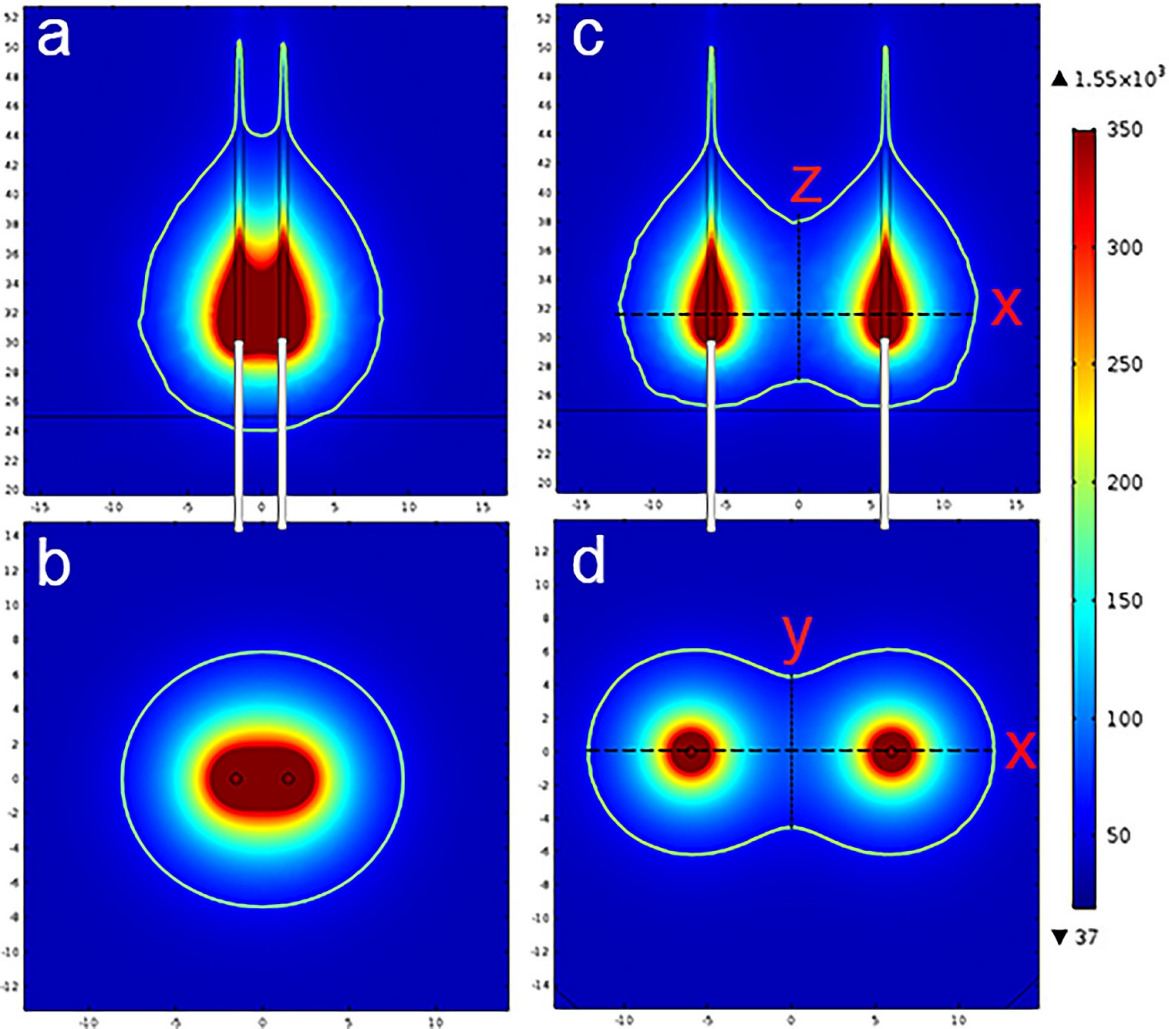
Temperature distributions after heating for 100 s with dual-fiber lasers with different spacing distances. (a, b) Spacing distance of 3 mm; (c, d) Spacing distance of 12 mm.

When fibers were spaced 3 mm apart (Fig 3A and 3B), the shape of the ablated region roughly resembled that of the ablation produced by a single-fiber laser, albeit with a larger size (the area of the dual-fiber ablated region was approximately 50% larger). As the spacing distance increasing, the ablated region between the two optical fibers gradually separated. When the spacing was 12 mm, as shown in Fig 3C and 3D, a large area between the two fibers was not ablated, and two independent teardrop shapes were formed.

**Fig 4:**
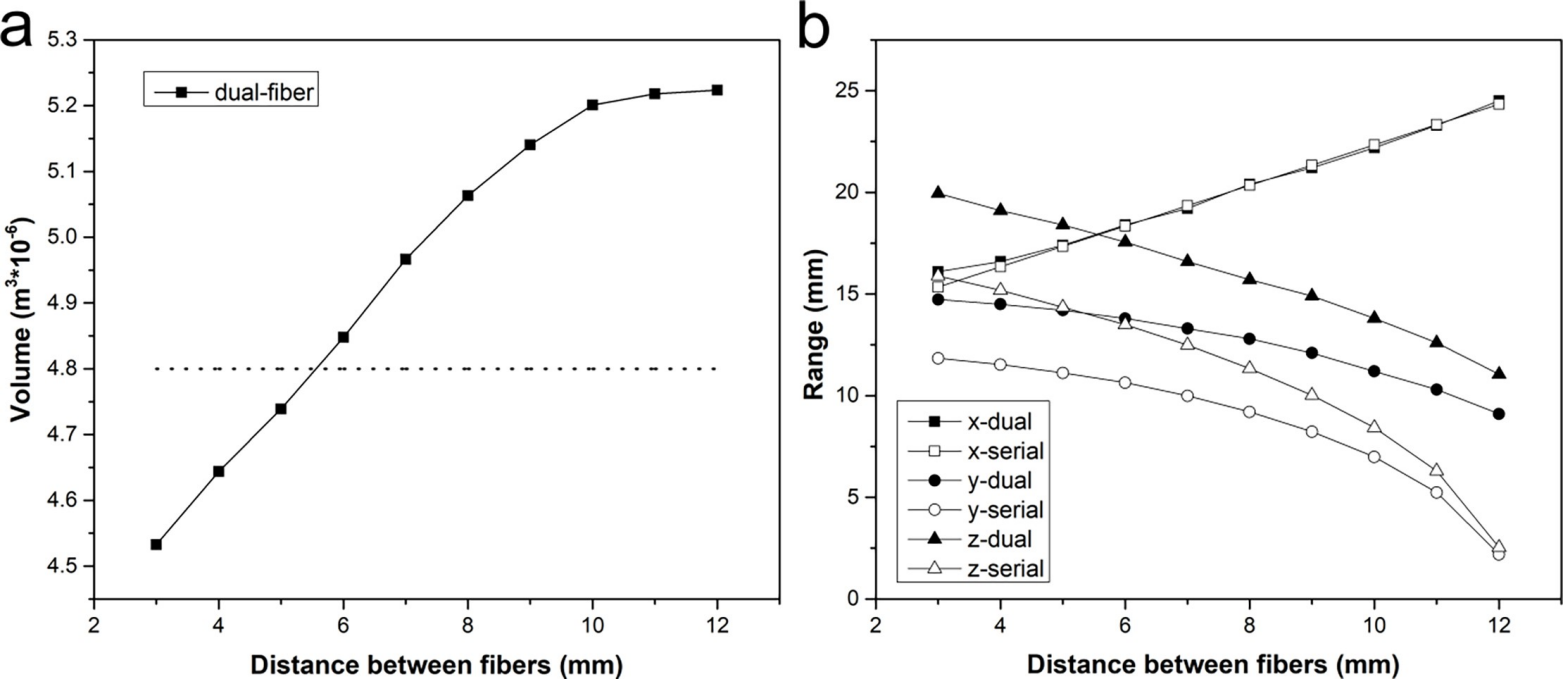
Dependence of both ablation volume and ablation range on the spacing between fibers. (a) The dependence of ablation volume on the spacing between the two fibers in dual-fiber ablation. The dotted line indicates the sum of the volumes of two single-fiber ablations, regarded as distance infinity. (b) The dependence of the maximum ablation range on the spacing between the two fibers along the x, y, and z directions.

To investigate the effect of the spacing distance between fibers on laser heating, spacing distances from 3 to 12 mm with increments of 1 mm were studied. Fig 4 shows the dependence of both the ablated volume (Fig 4A) and the maximum ablation range (Fig 4B) along the x, y, and z directions (shown in Fig 3C and 3D) on the spacing distance. As shown in Fig 4A, the ablation volume increased dramatically with the spacing distance when the two fibers were relatively close. The rate of increase gradually slowed when the two fibers were separated further, and the ablated volume reached its maximum. The dotted line represented the sum of the volumes of two single-fiber ablations (regarded as distance infinity), which was 4.8 × 10^−6^ m^3^. We found that the ablated volume resulting from a dual-fiber ablation was significantly greater than that of two single-fiber ablations. The maximum volume ablated by dual-fiber treatment was approximately 5.3 × 10^−6^ m^3^, which was 10.4% higher than that produced by the sum of two single-fiber treatments. When the two fibers were infinitely far from one another, the total ablation volume of the dual-fiber ablation would be the superposition of the two single-fiber ablations. Therefore, After the dual-fiber ablation volume reached its maximum value, it would gradually decline and eventually approach the dotted line. From Fig 4B, it can be observed that the incremental increases in the x direction were proportional to the spacing distance between the two fibers, with the exception of cases when the two fibers were very close. The x direction range for dual-fiber ablation was slightly larger than that for treatment with two serial single fibers at a distance of 3 mm, due to the overlapping thermal fields. The ablation ranges along the y and z directions decreased with an increase in the spacing distance. Compared with two serial-fiber treatments, the ablated ranges in these two directions were much larger for dual-fiber ablation. For example, at a distance of 7 mm, the ablation ranges increased by 33% in the y and z directions when dual-fiber treatment was used. With increased spacing distances this effect was more obvious, and dual-fiber ablation significantly increased the ablation range in the y and z directions.

### 3.2 *Ex vivo* experiments

**Fig 5:**
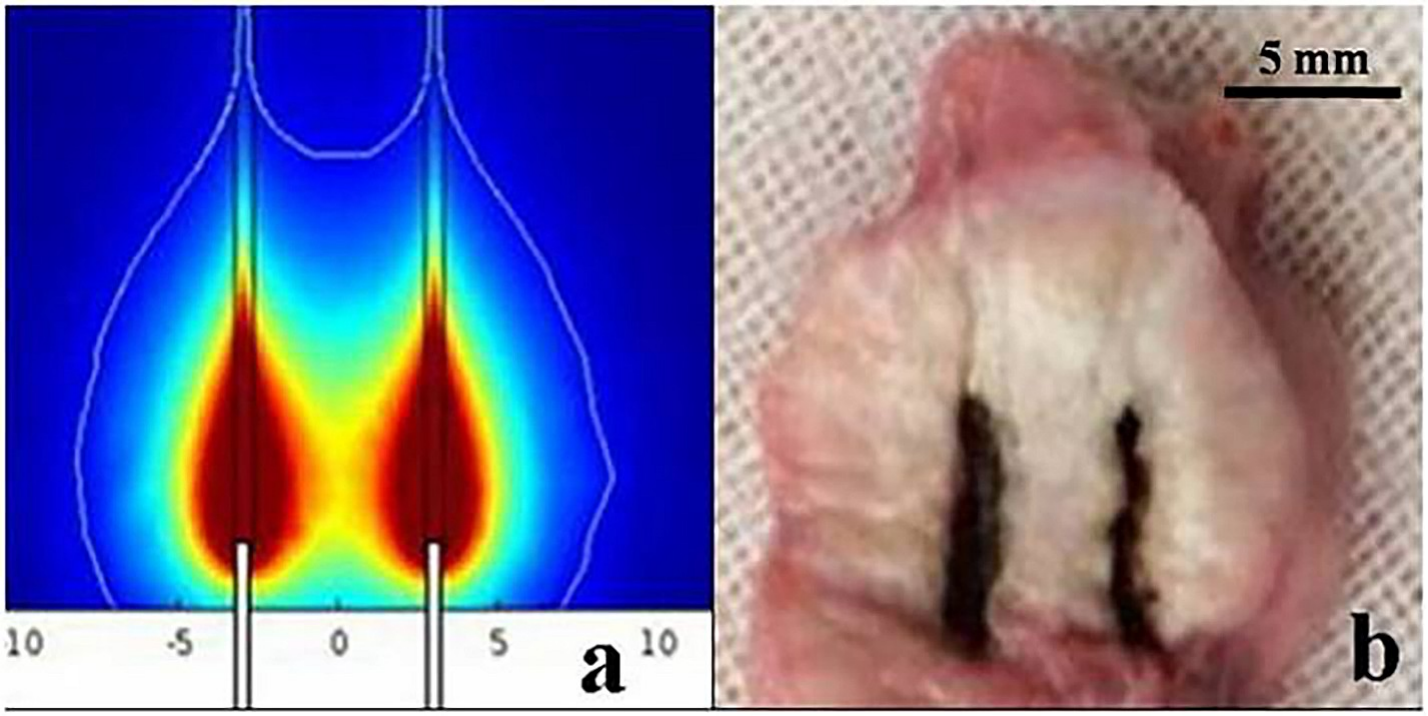
Comparison of the ablation zone in *ex vivo* experiment. (a) The shape of simulated ablation zone; (b) The shape of experimental zone.

To study the feasibility of using dual-fiber lasers for treating prostate cancer, *ex vivo* dual-fiber laser ablation experiments with a spacing of 6 mm were performed, and the results are shown in Fig 5. Normal prostate tissue was pink; in contrast, the ablated regions were pale white due to protein denaturation. Fig 5 compared the simulated ablative regions with the *ex vivo* ablations performed on canine prostate tissue. We found that the shapes of the cross sections of the maximum ablations in simulations and experiments resembled each other.

The transverse diameter (x) and anteroposterior diameter (z) of the ablation regions were measured and found to be 17±0.5 mm and 19±0.7 mm, respectively. The simulated values of the transverse diameter (x) and anteroposterior diameter (z) were found to be 16.30 mm and 18.11 mm, respectively, which were close to the experimental values. The maximum ablation area was defined by ImageJ. The maximum ablation areas in *ex vivo* tissue was 238 mm^2^ for the simulations and 259±67 mm^2^ for the experimental results.

### 3.3 *In vivo* experiments

**Fig 6:**
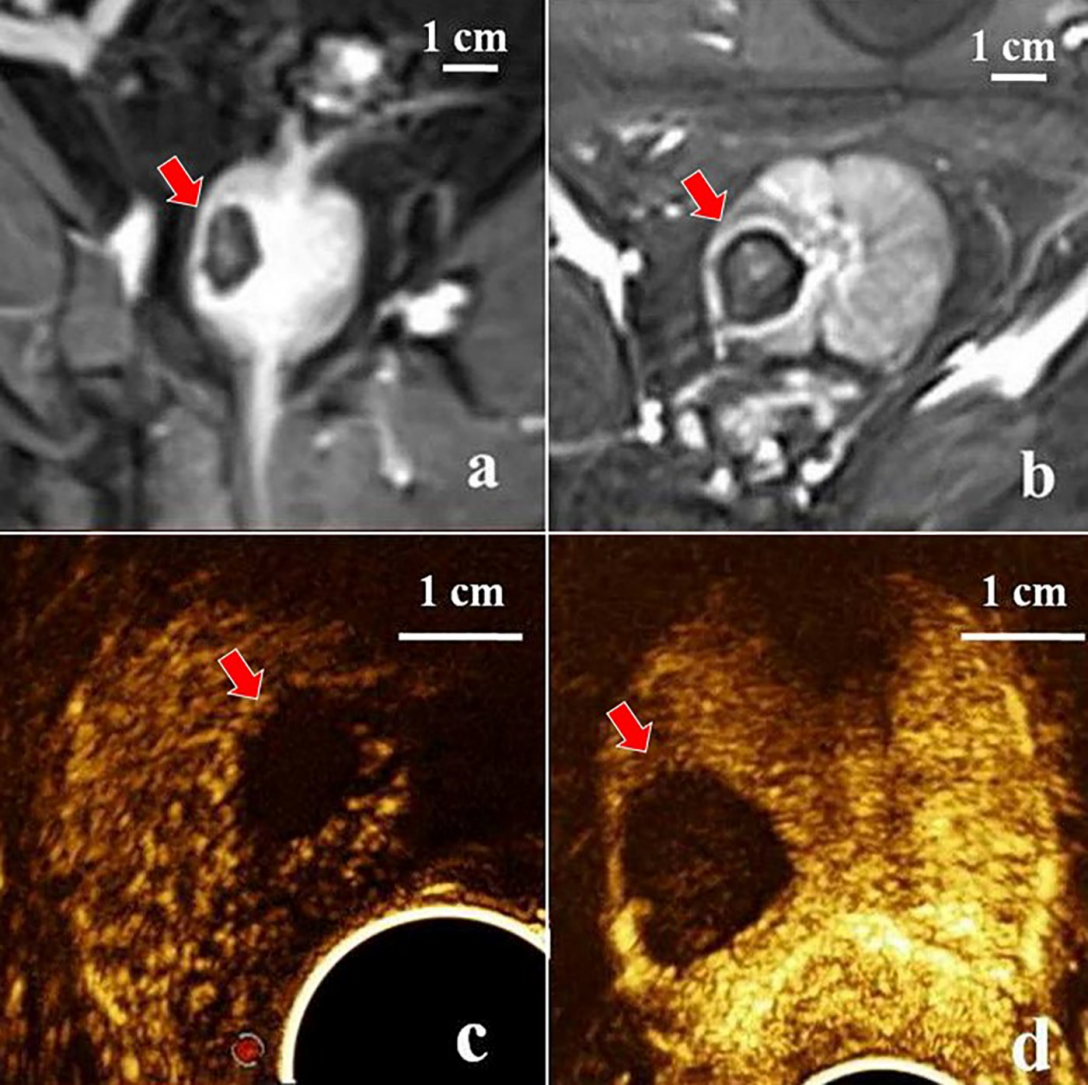
Comparison of the ablation zone in *in vivo* experiments. (a, b) Contrast-enhanced MRI imaging of the prostate after dual-fiber laser ablation. The ablation zone shows no filling of contrast agents (red arrow) and a weak signal; longitudinal (a) and transverse (b) sections are shown respectively; (c, d) CEUS imaging of the prostate after dual-fiber laser ablation. The ablation zone shows no filling of contrast agents (red arrow) and a weak signal; longitudinal (c) and transverse (d) sections are shown respectively.

Contrast-enhanced MRI and CEUS images of *in vivo* canine prostate tissues after dual-fiber laser ablation treatment (spacing distance 7 mm) are shown in Fig 6. As the tissues in the prostate ablation area were inactivated and lacked a blood supply, they were observed as filling defects, while the surrounding normal prostate tissues exhibited hyper-enhancement. Therefore, there was a distinct contrast between the two types of tissues. The three maximum radial lines of the ablation zone were measured via MRI and CEUS imaging, shown in Table 2.

**Fig 7:**
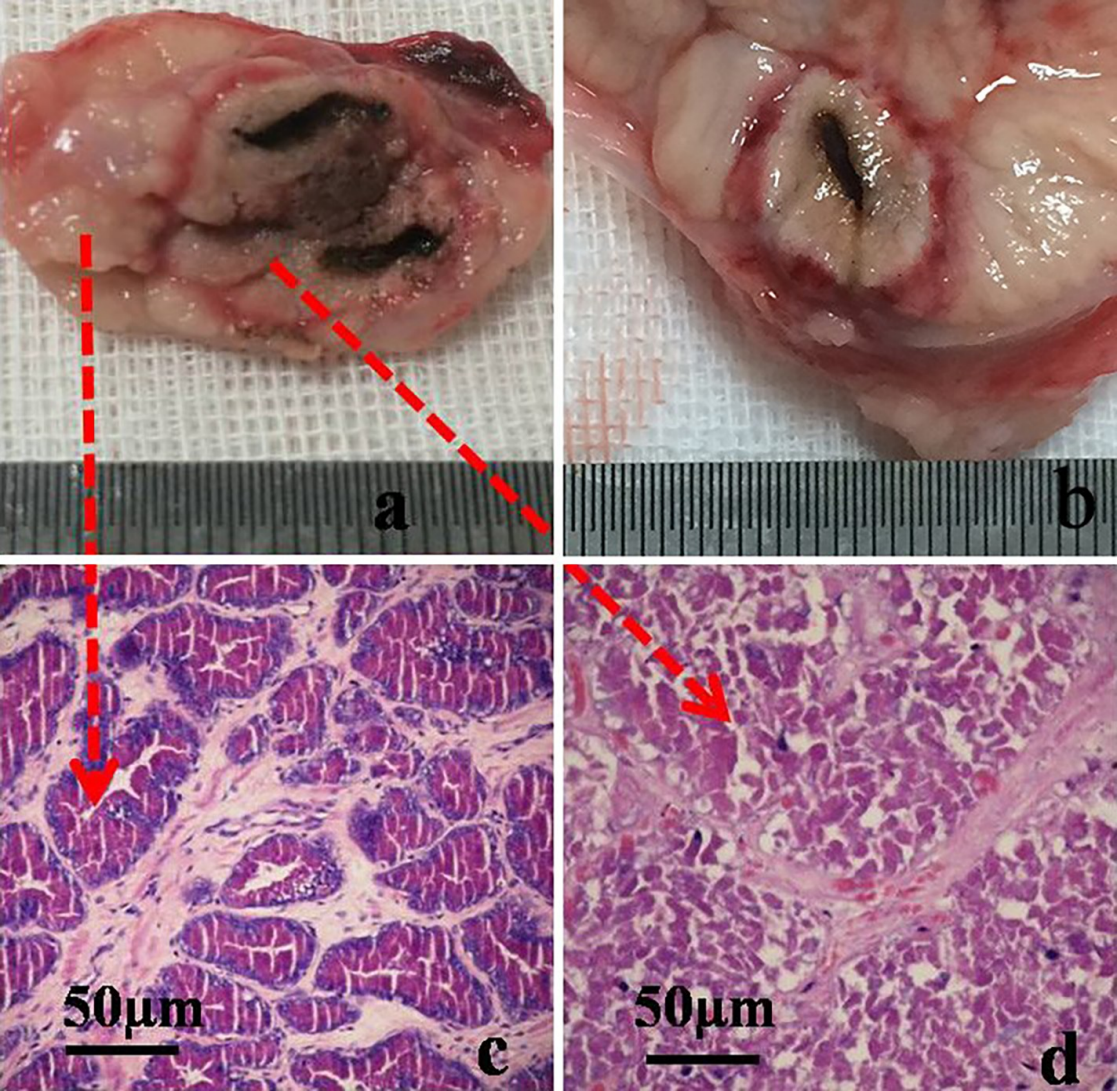
Comparison of *in vivo* experimental and simulated results. (a) A pathological section after dual-fiber laser ablation. Four regions were generally observed: a cavitation region containing the fibers, a carbonization region between the fibers, a pale region of inactivated tissue, and the edema cincture interfacing with normal tissues (b) A pathological section after single fiber laser ablation. (c) H&E staining of normal zone. (d) H&E staining of ablated tissue.

In excised pathological prostate tissues after treatment, four regions were generally observed: a cavitation region containing the fibers, a carbonization region between the fibers, a pale region of inactivated tissue, and the edema cincture interfacing with normal tissues (Fig 7A). As the edema cincture was clearly visible, the size of the ablation zone could also be accurately measured. The histopathological results revealed that the tissues within the ablation zone were all coagulation necrotic, and no area of incomplete ablation was observed between the two fibers (Fig 7C and 7D), which was consistent with the predicted results. Fig 7B showed a pathological section after single-fiber laser ablation, where the teardrop shape agreed well with the simulated results shown in Fig 2A. ImageJ was used to calculate the area of the lesions in the x-z plane in Fig 7A and 7B. The results showed that the lesion area resulting from dual-fiber treatment was 207.12 ± 10.13 mm^2^, while the simulated value was 239.07 mm^2^. The ablated area produced by single ablations was 104.10 mm^2^, while the simulated value was 125.66 mm^2^.

The three radial lines of the ablation obtained in the simulation were similar to the actual measurements determined by MRI, CEUS, and pathological examination (Table 2), thereby validating the model. When using two serial-fiber treatments, the simulated y range was 0.99 cm, and z range was 1.25 cm, which were much smaller than those using dual-fiber treatments. The maximum lesion area was 175.87 ± 8.70 mm^2^ in the transverse plane (x-y) and 163.81 ± 7.65 mm^2^ in the coronal plane (y-z) for *in vivo* experiments. The simulated values in the transverse and coronal planes were 191.99 mm^2^ and 164.27 mm^2^, respectively. The experimental area in the coronal plane was very similar to the simulated area, while the results for the transverse plane differed by 9.1%.

**Table 2:** Comparison of the simulated and *in vivo* measured ablation zones.

Group	Simulation x × z × y (cm)	MRI imaging x × z × y (cm)	CEUS x × z × y (cm)	Pathology x × z × y (cm)
**1**	1.92 × 1.66 × 1.32	1.82 × 1.59 × 1.35	1.82 × 1.72 × 1.61	1.71 × 1.45 × 1.20
**2**		1.70 × 1.45 × 1.12	1.78 × 1.65 × 1.55	1.65 × 1.32 × 1.13
**3**		1.95 × 1.70 × 1.40	2.01 × 1.75 × 1.53	1.80 × 1.58 × 1.25

## 4. Discussion

Both the *ex vivo* and *in vivo* experiments validated the model and confirmed that dual-fiber treatment can widen the treatment range in all three dimensions. The extensions of the ablation range in the y direction and z direction are significant. However, the ablation range in the x direction does not change significantly and is determined only by the distance between the two fibers. Dual-fiber treatment results in a larger ablation range than that resulting from two serial single ablations, the difference in which is primarily concentrated in the central region between the two fibers, which is shown by an increase in the y and z ranges. This difference is because the central region is at a relatively high temperature due to the heat transfer between the two fibers in the dual-fiber treatment, but the range at the edge region depends on heat transfer from the unilateral fiber, which is the same as that in a single-fiber ablation. Thus, compared to single-fiber ablation, dual-fiber ablation can achieve a greater ablation range in less time, especially in the areas between the two fibers.

For prostate ablations, it is important to protect the urethra and surrounding vessels. The curves in Fig 4 can be used in treatment planning, particularly for determining the spacing distance when using dual-fiber lasers, to prevent damage to normal tissues and organs. When the three dimensions of the tumor are known, an appropriate dual-fiber spacing can be chosen by referring to these curves. When the targeted tissue exceeds the maximum ablation range, treatment for more lesions may be considered.

This study also shows that both contrast-enhanced MRI and CEUS are effective methods to evaluate clinical treatment outcomes and can also be used for re-examination/follow-up. Ultrasound can guide the puncture needle into the prostate tissue for real-time positioning and dynamic imaging. MRI has a high soft tissue resolution and can provide multi-directional, multi-sequence imaging, resulting in a high image resolution when used in cases of prostate disease. CEUS and contrast MRI are capable of distinguishing lesions from normal tissue by differences in blood supply. Moreover, the two imaging modalities do not use radiation, can be operated repeatedly, and accurately measure the lesion range. Thus, MRI and CEUS are suitable choices for efficient evaluations and long-term follow-up.

In this study, a laser power of 5 W was chosen based on reports of clinical applications and *in vivo* experiments; laser ablation powers of 4 W to 15 W have been widely used [17–18]. However, the optimal laser power for ablation remains debatable, and various laser powers should be characterized in future studies. Based on the excellent agreement between the simulation and the experiments, even higher energies may be used, and more fibers may be introduced to simulate and study the ablation of larger areas. For follow-up studies of multi-focal prostate cancer, additional fibers can be used for ablation under the guidance of simulation results when dual-fiber ablation cannot cover all foci.

## 5. Conclusion

The results of the study presented herein indicate that dual-fiber laser ablation can remarkably increase the range of the ablation zone compared with single-fiber modes. This study has found the shape of the ablations created using dual-fiber laser ablation is quite different to the superposition of ablations using sequential single-fiber laser ablation. In addition, the shape of the ablation is dependent on the spacing distance between the two fibers. The results from the theoretical model and the experimental results were found to be in good agreement. The three-dimensional ablation ranges depended on the spacing distance between multiple fibers were also obtained which can be used for future treatment planning. The performed *in vivo* canine prostate experiments performed confirm the safety and feasibility of dual-fiber laser treatment for enlarging the ablation range, particularly in the y and z directions.
